# A case report of successful band ligation of bleeding anastomotic duodenal varix in an adolescent patient

**DOI:** 10.1002/jpr3.70017

**Published:** 2025-03-17

**Authors:** Lauren E. Hamilton, Joshua Carroll, Paul Tran

**Affiliations:** ^1^ University of Arizona College of Medicine Phoenix Glendale Arizona USA; ^2^ Phoenix Children's Hospital Phoenix Arizona USA; ^3^ Phoenix Children's Hospital University of Arizona College of Medicine Phoenix Glendale Arizona USA

**Keywords:** endoscopy, upper GI tract, varices

## Abstract

Ectopic varices are defined as portosystemic venous collaterals occurring in the gastrointestinal tract outside of the cardio‐esophageal region. Duodenal varices are not routinely encountered by pediatric gastroenterologists. At the time of this case report, there are no consensus guidelines on the management of bleeding duodenal varices in pediatric patients. This is a case of a 14‐year‐old young woman with a history of multi‐visceral transplantation due to short gut syndrome. The patient had developed duodenal varices near her transplant anastomosis, which were incidentally biopsied on endoscopy causing resultant bleeding that required endoscopic hemostasis. This case highlights the need for recognition of duodenal varices as a potential etiology of gastrointestinal bleeding in children and describes band ligation as an effective hemostatic modality.

## 1 INTRODUCTION

The prevalence of duodenal varices in pediatric patients with portal hypertension is limited. Varices are found less commonly in the duodenum than other regions of the gastrointestinal (GI) tract in this population. A 2019 study employed esophageal capsule endoscopy in children and young adults with portal hypertension for variceal screening or surveillance.^1^ The study found duodenal varices in only 6 out of 149 patients surveyed, reflecting 4% of the sample, compared with 17 gastric and 59 esophageal varices discovered.^1^ Literature describes children with portal hypertension and duodenal varices treated endoscopically, surgically, and/or with interventional radiology.^2^, ^3^, ^4^, ^5^

Singh et al. described a case of bleeding pediatric duodenal varices in 2022 in an 8‐year‐old boy who was 4 years postmultivisceral transplant.^2^ This patient was previously treated with endoscopic ultrasound‐guided sclerotherapy, which did not fully resolve bleeding. A transjugular intrahepatic portosystemic shunt procedure was subsequently performed, though it was ultimately unsuccessful.

This new case describes the successful endoscopic treatment of a patient similar to the one described by Singh et al., including a history of multivisceral transplant with bleeding anastomotic duodenal varices. In contrast, bleeding in this case was iatrogenic, incited by biopsies during a screening procedure. In a subsequent endoscopy, band ligation was used to achieve definitive hemostasis. This pediatric case highlights the importance of recognition of duodenal varices as a potential cause of GI bleeding in children, particularly those with history of multivisceral transplantation. It also describes variceal band ligation as an effective modality for lasting hemostasis of duodenal varices.

## 2 CASE REPORT

A 14 female with heterotaxy syndrome and jejunal atresia with secondary short bowel syndrome, multivisceral transplantation, portal hypertension, and posttransplant inflammatory bowel disease underwent a surveillance endoscopic procedure.

**Figure 1:**
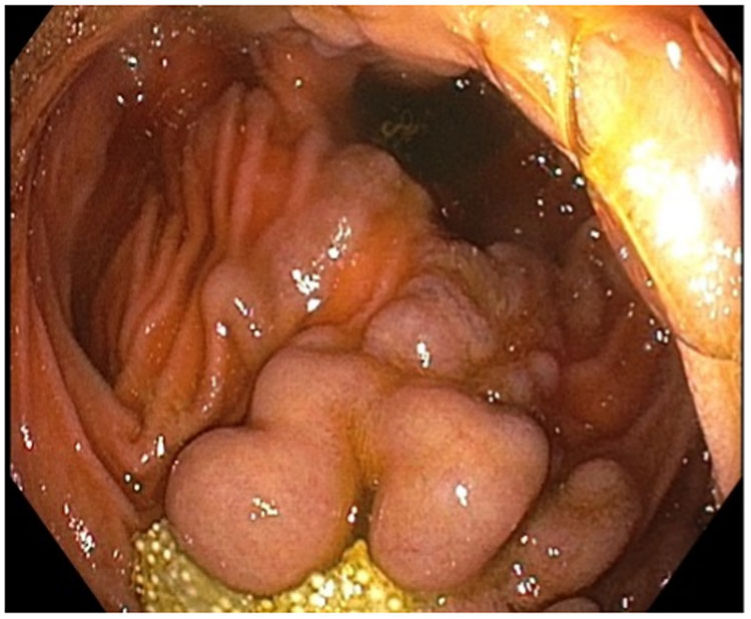
Initial endoscopy with mesalamine granules and polypoid masses in the area of duodenal anastomosis (duodenal varices).

During esophagogastroduodenoscopy and colonoscopy for staging of her inflammatory bowel disease, multiple unusual duodenal polypoid lesions were identified (see Figure 1) and biopsied. This led to immediate bleeding and formation of a mass spanning the entire duodenal lumen. Given the rapid formation of the mass, there was concern for hematoma versus blood clot. The patient was subsequently admitted for monitoring overnight. Over the next 24 h, despite being asymptomatic and without hematemesis or hematochezia, her hemoglobin trended down from 9.6 to 7.6 g/dL. On further review of the patient's chart, duodenal varices had been noted on previous endoscopies. With increased concern for variceal bleeding, she was transfused with packed red blood cells, octreotide was initiated, and repeat endoscopy was urgently scheduled.

**Figure 2:**
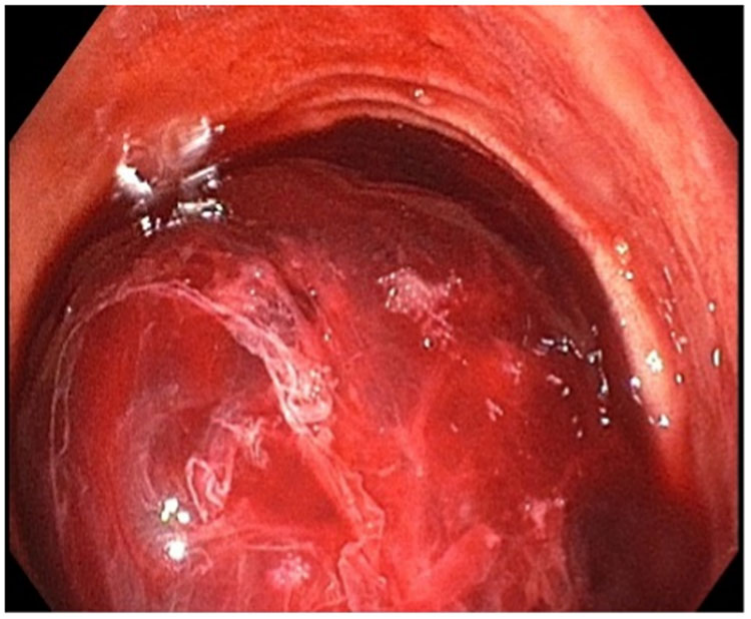
Rapid formation of lumen‐filling clot after biopsy on initial endoscopy.

**Figure 3:**
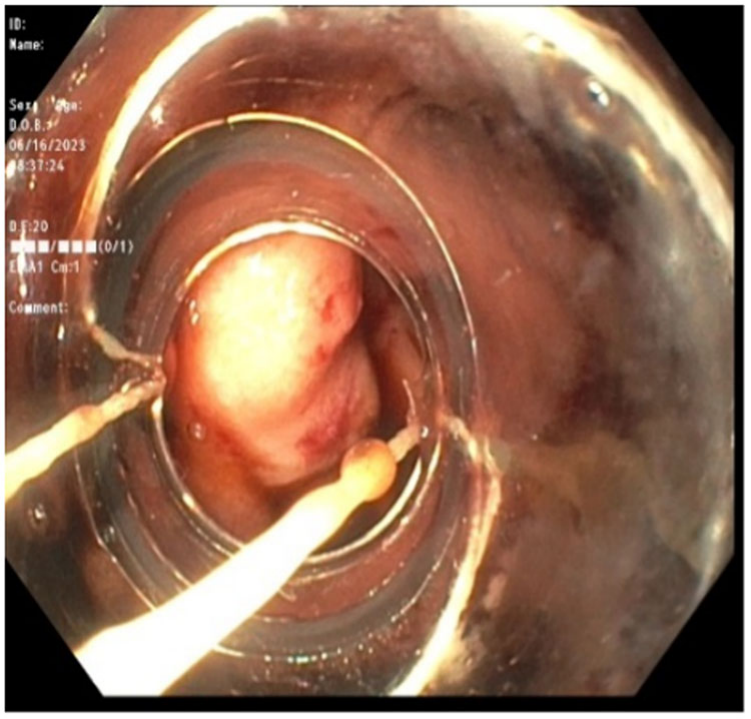
Following clot removal, duodenal varix visualized with prominent biopsy markings. Effective decompression with placement of a single band.

During repeat endoscopy, dark blood was suctioned from the stomach. On advancement of the gastroscope, a sizable clot was visualized in the duodenum (see Figure 2). Following removal of the blood clot with a combination of suction and snare transection, a nonbleeding varix with prominent biopsy markings was noted in the area of the duodenal anastomosis. A single band was deployed with effective hemostasis (see Figure 3). Following the procedure, the patient was weaned off octreotide over the following 48 h, with stable hemoglobin and no evidence of further bleeding. Her diet was advanced and she was discharged home. Hemoglobin continued to remain stable in subsequent clinic visits with no recurrence of symptoms.

## 3 DISCUSSION

This case highlights iatrogenic variceal bleeding during endoscopy necessitating hospitalization and repeat endoscopic intervention. Bleeding was due to a failure to correctly identify varices in this pediatric patient. Given the rarity and unfamiliar location outside of the esophagus, ectopic varices may not be accurately identified by an endoscopist.^5^, ^6^ Identification is critical, given the mortality rate for bleeding duodenal varices has been found to be as high as 13.9% in a 2020 systematic review of adult patients.^7^ Ectopic varices are typically described in the context of portal hypertension but can also present due to congenital anomalous portosystemic anastomoses, arteriovenous fistulae, abnormal vessel structure, or in relation to thromboses of intra‐abdominal vessels.^8^ Accurate recognition of duodenal varices may be increasingly important as children with portal hypertension and intestinal transplants are living longer. If available, endoscopic ultrasound may be useful in the identification of duodenal varices.^9^

There are a variety of approaches to duodenal variceal bleeding described in adult patients, including endoscopic, surgical, and interventional radiologic procedures. Primary endoscopic therapies include injection sclerotherapy and band ligation. Historically, the largest sample studies available on endoscopic treatments for bleeding duodenal varices demonstrate these treatments to be successful, however they were limited by small sample sizes (under 50 patients) and without comparison groups.^10^, ^11^ A 2021 systematic review of 156 patients in Jiangxi, China assessed effectiveness and safety of endoscopic treatment for duodenal variceal bleeding. Endoscopic therapeutic modalities included endoscopic band ligation, endoscopic injection sclerotherapy, endoscopic tissue adhesive, and combination treatment. The study found a rebleeding rate of 8.9% and a mortality rate of 13.9%, which are considerably lower estimates than previous reviews.^7^ The review found treatment success, rebleeding, and mortality not statistically significantly different between groups. The authors suggested endoscopic intervention as a feasible, well‐tolerated, and effective modality for duodenal variceal bleeding but called for more research into the comparison of the different endoscopic modalities.^7^

This patient had noted history of anastomotic varices on prior endoscopies, with prior sclerotherapy used successfully, though at a much younger age. After successful endoscopic visualization of the duodenal varices, the team discussed band ligation versus sclerotherapy, opting for the former given concern for potential stricture formation with repeated sclerotherapy. Banding in this case, achieved lasting hemostasis, with no recurrent bleeding or anemia, both immediately postoperatively and on follow‐up.

In comparison to the most recently reported case in the UK, our patient experienced iatrogenic bleeding, rather than spontaneous bleeding in the setting of portal hypertension. Both cases included a history of ulcerations and varices of anastomotic vessels in the setting of the joined graft and native bowel. In the case of this 14‐year‐old female, banding proved to be an effective modality for effective hemostasis. This approach may be a possible alternative to or adjuvant therapy to sclerotherapy, but further research is needed to determine best treatment of bleeding ectopic varices in pediatric patients.

## CONFLICT OF INTEREST STATEMENT

Paul Tran is a consultant for EvoEndo, Inc which is unrelated to this manuscript. The other authors declare no conflicts of interest.

## ETHICS STATEMENT

Before writing this case report, informed consent and approval were obtained from the patient's parents.
